# Hyperhomocysteinemia: A Predictor of Microvascular Complications in Type 2 Diabetes Mellitus

**DOI:** 10.7759/cureus.89826

**Published:** 2025-08-11

**Authors:** Shali S, Gopalakrishna Pillai, Namitha Salim, Sheejamol VS

**Affiliations:** 1 Internal Medicine, Amrita Institute of Medical Sciences and Research Center, Kochi, IND; 2 General Medicine, Amrita Institute of Medical Sciences and Research Center, Kochi, IND; 3 Biostatistics, Amrita Institute of Medical Sciences and Research Center, Kochi, IND

**Keywords:** diabetes-related peripheral neuropathy, diabetic microvascular complications, diabetic retinopathy, diabtetic nephropathy, homocysteine levels, type 2 diabetes mellitus

## Abstract

Background

Diabetes mellitus (DM) is a very crippling illness that affects a large number of people around the world. Raised homocysteine levels are linked to a number of harmful health issues. It is possible that homocysteine contributes to the development of diabetic microangiopathy because it is involved in a complex and dynamic system of vascular damage and repair. Given that hyperhomocysteinemia can be treated nutritionally, this highly debated topic has significant scientific and therapeutic ramifications. Hence, the purpose of this study is to ascertain the correlation between hyperhomocysteinemia and microvascular complications in type 2 diabetes mellitus (T2DM). Thus, the expected outcomes of this study will be the discovery of a novel predictor of diabetic microangiopathy: elevated homocysteine.

Aim

This study aims to evaluate the levels of homocysteine in diabetes mellitus patients with and without microvascular complications and to find the association between hyperhomocysteinemia and microvascular complications of type 2 diabetes mellitus.

Method

This is a prospective observational study involving all type 2 diabetes mellitus patients from outpatient and inpatient settings from general medicine, endocrinology, nephrology, ophthalmology, and podiatry departments of Amrita Institute of Medical Sciences (AIMS), Kochi, conducted between February 2022 and June 2024. The study population is categorized as patients with complications and controls without complications. Fasting plasma homocysteine levels are compared. Based on the results of the mean and standard deviation (SD) of fasting plasma homocysteine among diabetic patients with complications (9.4±3.1) and without complications (7.4±2.8), as mentioned in an earlier publication, with 90% power and 95% confidence, the minimum sample size is approximately 46 participants in each group, totaling 92.

Results

Among the 92 study participants who are patients with diabetes mellitus, 46 were without any of the major or minor complications of diabetes mellitus, grouped as Group 1, and the remaining 46 had at least one of the minor complications of diabetes mellitus, grouped as Group 2. In the diabetic retinopathy population, the mean serum homocysteine was 17.1±5.4 µmol/L, while in those who do not have diabetic retinopathy, it was 9.8±4.3 µmol/L, with a significantly higher p-value of <0.001. In those with diabetic neuropathy, the mean serum homocysteine was 17.0±5.5 µmol/L, while in those without diabetic neuropathy, it was 9.9±4.3 µmol/L, which is significantly higher, with a p-value of <0.001. In the diabetic nephropathy population, the mean serum homocysteine was 17.7±4.9 µmol/L, while in those without diabetic nephropathy, it was 9.7±4.2 µmol/L, which is significantly higher, with a p-value of <0.001. Using the receiver operating characteristic (ROC) curve, the area under the curve (AUC) was 0.9, with a 95% confidence interval (CI) of 0.83-0.97. Serum homocysteine is showing higher AUC; hence, it can be used as a predictor of complications (p<0.001), and the cutoff is taken as 11.8 µmol/L, with a sensitivity of 83% and a specificity of 87%.

Conclusion

This study was able to draw a significant association between high levels of plasma homocysteine and microvascular complications such as diabetic neuropathy, nephropathy, and retinopathy.

## Introduction

Hyperglycemia is the common trait shared by a set of metabolic illnesses collectively referred to as diabetes mellitus (DM). Many forms of diabetes mellitus are brought on by a complicated interplay between heredity and exogenous factors from the environment. With different kinds of diabetes mellitus, there may be a variety of causes for hyperglycemia, but they frequently include reduced insulin secretion and increased insulin resistance. In patients with diabetes mellitus, there will be alterations of metabolism in other pathways, which in turn may lead to changes that are extremely taxing on the diabetic patient, as well as the healthcare system. Adult blindness, non-traumatic lower extremity amputations, and end-stage renal disease (ESRD) are all complications of DM. Cardiovascular illnesses are also predisposed by it [1].

Raised homocysteine levels are linked to a number of harmful health issues. Homocysteine is an amino acid that has an inverse relationship with folate and vitamin B12. Hence, this study aims to find the possible involvement of homocysteine in diabetes with microvascular complications.

More epidemiological research on the relationship between homocysteine and diabetes is advised to manage hyperhomocysteinemia and the ensuing rising burden of disease, particularly diabetes [2-4]. It is possible that homocysteine contributes to the development of diabetic microangiopathy because it is involved in a complex and dynamic system of vascular damage and repair. Given that hyperhomocysteinemia can be treated nutritionally, this highly debated topic has significant scientific and therapeutic ramifications. With the primary goal of lowering homocysteine levels through diet in order to reduce the occurrence of microangiopathy in patients with type 2 diabetes mellitus, the purpose of this study is to ascertain the correlation between hyperhomocysteinemia and microvascular complications in type 2 diabetes mellitus. Thus, the expected outcomes from this study will be the identification of elevated homocysteine as a novel predictor of diabetic microangiopathy.

Aim of the study

This study aims to evaluate the levels of homocysteine in diabetes mellitus patients with and without microvascular complications and to find the association between hyperhomocysteinemia and microvascular complications of type 2 diabetes mellitus.

Primary objective

The primary objective of this study is to compare fasting plasma homocysteine levels between type 2 diabetes mellitus patients with microvascular complications and type 2 diabetes mellitus patients without microvascular complications and to find the association between hyperhomocysteinemia and microvascular complications of type 2 diabetes mellitus.

Secondary objective

This study also aims to find the cutoff value of fasting plasma homocysteine levels above which the risk of microangiopathy increases.

## Materials and methods

This is a prospective observational study involving all type 2 diabetes mellitus patients from outpatient and inpatient settings from general medicine, endocrinology, nephrology, ophthalmology, and podiatry departments of Amrita Institute of Medical Sciences (AIMS), Kochi, conducted between February 2022 and June 2024. This research has been approved by both the dissertation review committee and the ethical committee of the institution (approval number: ECASM-AIMS-2023-097).

Inclusion criteria

Type 2 diabetes mellitus patients of either sex who are between the ages of 18 and 80 years were included in this study.

Exclusion criteria

We excluded patients with fever, urinary tract infections, and acute kidney injury; those who had received folates, cyanocobalamin, and pyridoxine in the previous 90 days; those who had been administered metformin, carbamazepine, phenytoin, methotrexate, and diuretics in the last 30 days; patients with non-diabetic nephropathy-causing renal failure, pernicious anemia, hypothyroidism, and psoriasis, causing dysregulation of metabolism of folates, cyanocobalamin, and pyridoxine; smokers or reformed smokers who had quit smoking for not more than 365 days; patients who have undergone ileal or jejunal resection or with vitamin B12 and folate deficiency; and those with any macrovascular complications of diabetes mellitus.

Sample size

Based on the results of the mean and standard deviation (SD) of fasting plasma homocysteine among diabetic patients with complications (9.4±3.1) and without complications (7.4±2.8) observed in an earlier publication by Idzior-Walus et al. [5] and with 90% power and 95% confidence, the minimum sample size comes to 46 in each group, totaling 92 [5].

Statistical analysis

Statistical analysis was done using the IBM SPSS Statistics version 20 for Windows (SPSS Inc., Chicago, Ill). The results are given as mean ± SD or median (Q1-Q3) for continuous variables and frequency (percentage) for categorical variables. The normality of the data were checked using the Kolmogorov-Smirnov test. The Pearson Chi-square test was used to find the association between two categorical variables. To test for statistical significance in the mean levels of serum homocysteine, serum creatinine, glomerular filtration rate (GFR), HbA1C, and age between the two groups, an independent samples t-test was applied. To test for a statistically significant difference in the median duration between two groups, the Mann-Whitney U test was applied. Receiver operating characteristic (ROC) curve analysis was used to calculate the area under the curve (AUC) to find the optimal cutoff of serum homocysteine to predict complications, and its sensitivity and specificity were estimated. A p-value of <0.05 was considered statistically significant. All tests of statistical significance were two-tailed. Multiple logistic regression was used to detect the independent effect of risk factors on type 2 diabetes mellitus with complications such as diabetic retinopathy, nephropathy, and neuropathy.

## Results

Among the 92 study participants with diabetes mellitus, 46 were without any of the major or minor complications of diabetes mellitus, grouped as Group 1, and the remaining 46 had at least one of the minor complications of diabetes mellitus, grouped as Group 2.

Table 1 shows that there is no statistically significant difference in the mean age between the two groups (p=0.14). Likewise, there is no statistically significant association between gender and DM complication (p=0.11). The median duration of diabetes mellitus in patients with and without complications was compared and were found to be statistically significant, with a p-value of <0.001. HbA1C levels were compared between the two groups, but the difference was not statistically significant (p=0.25). A statistically significant difference was observed in the creatinine values among the two groups, with significantly higher creatinine in Group 2 (p<0.001). The difference in GFR values among the two groups was statistically significant, with significantly lower GFR in Group 2 (p<0.001). Regarding serum homocysteine values among the two groups, there was a statistically significant difference, with significantly higher serum homocysteine in Group 2 (p<0.001).

**Table 1:** Patient characteristics Age is represented as mean±SD, gender is represented as numbers and percentages within brackets, duration is presented as mean±SD in number of years, HbA1C is represented as mean±SD in percentage (%), serum creatinine is represented as mean±SD in mg/dL, GFR is represented as mean±SD in mL/minute, and serum homocysteine is represented as mean±SD in µmol/L. The Mann-Whitney U test is applied to test the statistical significance of duration of diabetes mellitus between Group 1 and Group 2, Chi-square test is applied to test the statistical significance of gender (male and female) in diabetes mellitus between Group 1 and Group 2, and independent t-test is applied to test the statistical significance of age, HbA1C, serum creatinine, GFR, and serum homocysteine between Group 1 and Group 2. A p-value of <0.05 was considered statistically significant. SD: standard deviation, GFR: glomerular filtration rate, S. creatinine: serum creatinine, S. homocysteine: serum homocysteine

Variable		Value		Statistical values	p-value
		Group 1	Group 2		
Age (years)	Mean±SD	58.24±13.6	54±12.52	t=1.48	0.14
Male	Number (%)	28 (60.9%)	35 (76.1%)	Chi-square=2.47	0.11
Female	Number (%)	18 (39.1%)	11 (23.9 %)		
Duration (years)	Median (Q1-Q3)	17 (10-25)	10 (5-15)	Z=-4.327	<0.001
HbA1C (%)	Mean±SD	7.6±1.6	8.02±1.6	t=1.157	0.25
S. creatinine (mg/dL)	Mean±SD	0.7±0.2	1.7±1.2	t=5.164	<0.001
GFR (mL/minute)	Mean±SD	68±6	55±14	t=-5.506	<0.001
S. homocysteine (µmol/L)	Mean±SD	8.96±2.6	17.1±5.6	t=8.775	<0.001

**Figure 1:**
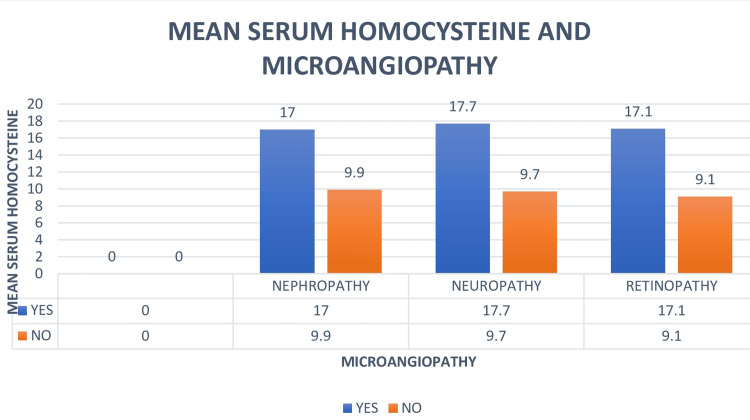
Comparison of mean serum homocysteine with individual microvascular complications Serum homocysteine is compared with different microvascular complications and is represented as mean±SD in µmol/L. SD: standard deviation

There are significantly higher values of serum homocysteine in the diabetic retinopathy population than in the population without diabetic retinopathy, with a p-value of <0.001. There are significantly higher values of serum homocysteine in the diabetic nephropathy population than in the population without diabetic nephropathy, with a p-value of <0.001. There are significantly higher values of serum homocysteine in the diabetic nephropathy population than in the population without diabetic nephropathy, with a p-value of <0.001 (Table 2 and Figure 1).

**Table 2:** Comparison of serum homocysteine with individual microvascular complications The number of subjects with or without microvascular complications is represented as numbers and percentages within the brackets. Serum homocysteine is compared with different microvascular complications and is represented as mean±SD in µmol/L. An independent t-test is applied to test the statistical significance of serum homocysteine between the groups with and without individual microvascular complications such as diabetic retinopathy, nephropathy, and neuropathy. A p-value of <0.05 was considered significant. SD: standard deviation

Complications	Yes/no	Number (%)	Mean±SD (µmol/L)	Statistical values	p-value
Diabetic retinopathy	Yes	40 (43%)	17.1±5.4	t=7.105	<0.001
	No	52 (57%)	9.8±4.3		
Diabetic neuropathy	Yes	38 (41%)	17.7±4.9	t=8.279	<0.001
	No	54 (59%)	9.7±4.2		
Diabetic nephropathy	Yes	40 (43%)	17.0±5.5	t=7.239	<0.001
	No	52 (57%)	9.9±4.3		

The multiple logistic regression analysis revealed that patients with hyperhomocysteinemia have higher risks of developing neuropathy (odds ratio (OR): 1.28, 95% (confidence interval) CI: 1.093-1.486, p=0.002), diabetic nephropathy (OR: 1.57, 95% CI: 1.168-2.098, p=0.003), and diabetic retinopathy (OR: 1.18, 95% CI: 1.007-1.396, p<0.001). These results suggest that hyperhomocysteinemia is associated with an increased risk of diabetic neuropathy, nephropathy, and retinopathy in the study population. The p-value for the association with neuropathy, nephropathy, and retinopathy is statistically significant (Table 3).

**Table 3:** Association between hyperhomocysteinemia and diabetic nephropathy, neuropathy, and retinopathy

Parameters	Odds ratio	95% confidence interval of or		p-value
Nephropathy	1.57	1.168	2.098	0.003
Neuropathy	1.28	1.093	1.486	0.002
Retinopathy	1.18	1.007	1.396	<0.001

**Figure 2:**
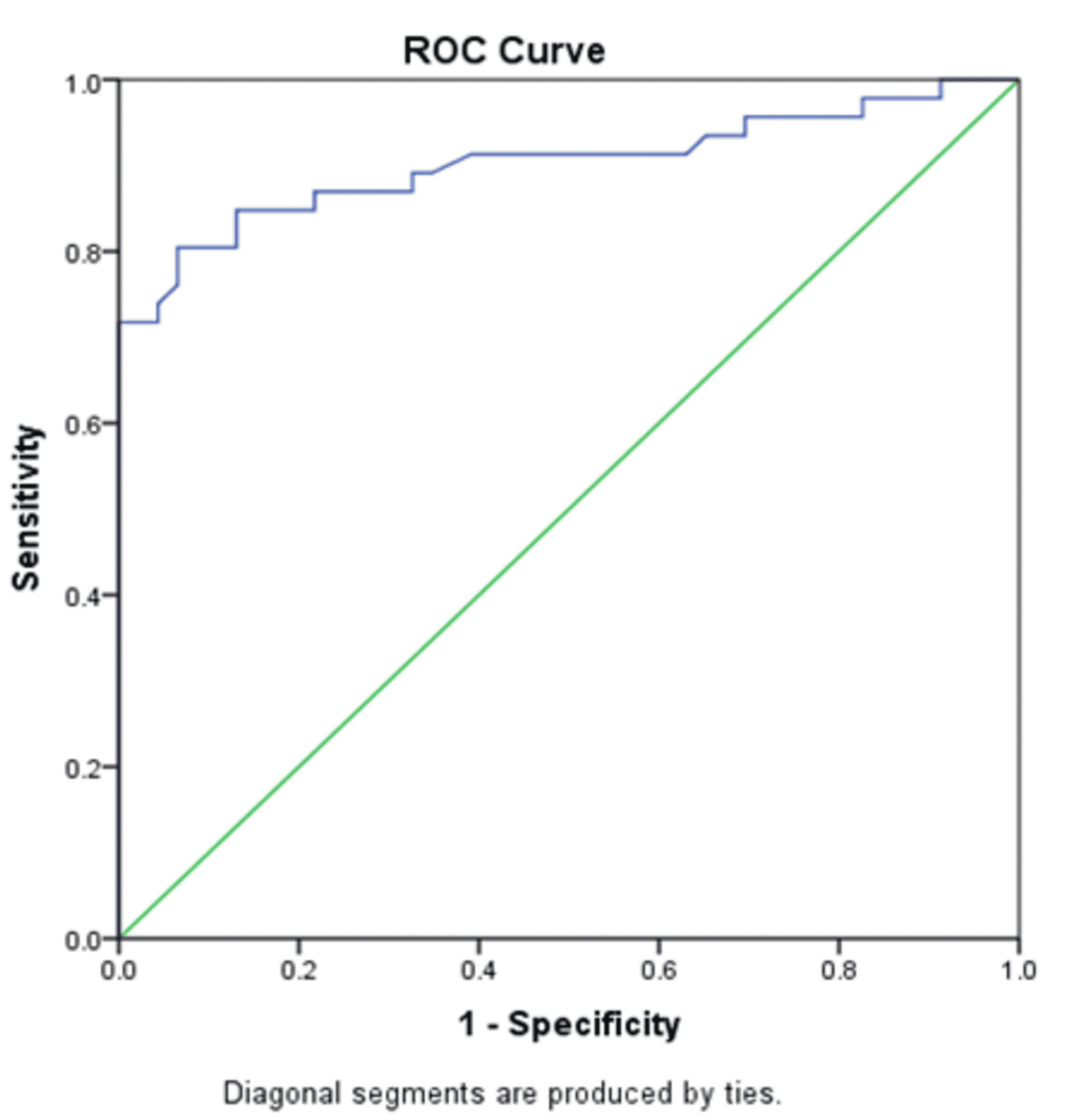
ROC curve for finding the sensitivity and specificity of serum homocysteine as a novel predictor of microangiopathy ROC: receiver operating characteristic

Using the ROC curve, the area under the curve (AUC) is 0.9, with a 95% confidence interval of 0.83-0.97. Serum homocysteine shows higher AUC; hence, it can be used as a predictor of complications (p<0.001), and the cutoff is taken as 11.8 µmol/L, with a sensitivity of 83% and a specificity of 87% (Table 4 and Figure 2). 

**Table 4:** Results of ROC curve The test result variable(s): serum homocysteine has at least one tie between the positive actual state group and the negative actual state group. Statistics may be biased. ^a^Under the nonparametric assumption ^b^Null hypothesis: true area = 0.5 ROC curve analysis was applied to find the optimal cutoff of serum homocysteine. A p-value of <0.05 was considered statistically significant. ROC: receiver operating characteristic

Test result variable(s): serum homocysteine				
Area	Standard error^a^	p-value	Asymptotic 95% confidence interval	
			Lower bound	Upper bound
0.903	0.035	<0.001	0.834	0.971

## Discussion

The sulfur-containing amino acid homocysteine has been found to be associated with coronary vascular disease. This association between homocysteine and the long-term effects of diabetes mellitus could be explained by either an indirect influence on endothelial cell methylation or a direct detrimental effect on vascular endothelial cells [2-4]. High homocysteine levels have been associated with an increased risk of stroke and other cardiovascular events [6-9]. The direct toxic effect of homocysteine, which causes vasculopathic accident events, is attributed to microdamage of the inner lining cells of the vascular tree. This prospective research aims to determine whether insulin resistance-related diabetes and its associated microvascular complications are linked to hyperhomocysteinemia.

The study group accommodates 50% of patients without DM without complications (Group 1) and 50% of patients with complications (Group 2), with p-values of 0.14 and 0.11, respectively. There is no statistically significant difference in age or gender between the two groups. In the study by de Luis et al., they noted that the majority of patients were 64.6±8.6 years old [7].

There was a statistically significant difference in creatinine values between the two groups, with significantly higher creatinine in Group 2, with a p-value of <0.001. Hence, serum creatinine levels are significantly higher in the group with diabetes and microvascular complications. This is in accordance with the study done by Li et al. [10].

Of 533 patients with type 2 diabetes in the study conducted by Soedamah-Muthu et al., 267 (50%) were female patients and 267 (50%) were male patients [11]. The mean duration of diabetes in this study was 14.6±9.6 years. The median duration of diabetes mellitus in patients without complications (Group 1) was 10 (5-15) years, while the median duration of diabetes mellitus in patients with complications (Group 2) was 17 (10-25) years. The difference between the two groups were found to be statistically significant, with a p-value of <0.001. The prevalence of microvascular complications in diabetes increases with the duration of diabetes. In the study by Soedamah-Muthu et al., the median duration of diabetes was 22 years; in the group with complications (Group 2), it was 24.6±9.1 years, and in the group without complications (Group 1), it was 15.4±6.6 years [11].

There is no statistical significance in HbA1C among the two groups (p=0.25). Similar results were found for HbA1C between people with type 1 diabetes mellitus with diabetic retinopathy and those without diabetic retinopathy in the study conducted by Saeed et al. [12].

There was a statistically significant difference in GFR values between the two groups, with significantly lower GFR in Group 2, with a p-value of <0.001. Hence, GFR is significantly lower in the group with diabetes and microvascular complications. However, a reverse causation is implied here as reduced GFR can decrease the rate of clearance of serum homocysteine; hence, it is a confounder. However, the study by Li et al. proved that hyperhomocysteinemia is non-linearly associated with increased risk of diabetic nephropathy [10].

Serum homocysteine levels were significantly higher in the diabetic retinopathy population than in the population without diabetic retinopathy, with a p-value of <0.001. According to the study by Saeed et al., there was an association between fasting levels of homocysteine and diabetic retinopathy in type 1 diabetes mellitus patients [12]. The group with retinopathy had higher fasting homocysteine levels (8.75±1.9 versus 7.69±1.6 μmol/L, p<0.05) than the group without retinopathy. In the group with diabetic retinopathy, microalbuminuria was more common and was associated with homocysteine levels (p<0.05).

Serum homocysteine levels were significantly higher in the diabetic neuropathy population than in the population without diabetic neuropathy, with a p-value of <0.001. According to the study by González et al. [13] and Lin et al. [14], plasma homocysteine concentrations were significantly higher in patients with high-grade peripheral neuropathy (9.34±2.27 and 11.72 ± 3.97 versus 13.54±5.6, p=0.001).

Serum homocysteine levels were significantly higher in the diabetic nephropathy population than in the population without diabetic nephropathy, with a p-value of <0.001. This is in accordance with the study conducted by Ye et al., in which 55 patients with type 2 diabetes mellitus with diabetic nephropathy and 51 patients with type 2 diabetes mellitus without diabetic nephropathy were studied, and their serum homocysteine was measured. It was found that the serum levels of homocysteine were 15.49±5.40 and 9.23±3.15 μmol/L for diabetic nephropathy and type 2 diabetes mellitus without diabetic nephropathy patients, respectively, with a statistical difference (t=7.21, p<0.001). In the diabetic nephropathy group, the serum homocysteine levels for patients with hyperfiltration, intermittent proteinuria, microalbuminuria, macroalbuminuria, and uremia were 10.99±2.57, 13.90±2.86, 15.38±4.77, 18.98±4.36, and 23.31±5.22 μmol/L, respectively, which indicated that serum homocysteine levels were higher in diabetic nephropathy patients than in T2DM patients and correlated with patients' renal damage [15].

Serum homocysteine was compared between the two groups, and it was found to have a mean of 8.96±2.6 µmol/L in Group 1 and 17.1±5.6 µmol/L in Group 2. There was a statistically significant difference in serum homocysteine values among the two groups, with significantly higher serum homocysteine in Group 2, with a p-value of <0.001. In accordance with the study by Barry et al., the analysis of neuropathy and nephropathy frequencies among type 2 diabetic patients, stratified by homocysteine concentrations, demonstrated a notably higher prevalence of diabetic nephropathy in patients with hyperhomocysteinemia compared to those with normohomocysteinemia (23.07% versus 8.75%, p=0.052). Similarly, diabetic neuropathy exhibited a significantly greater frequency in patients with hyperhomocysteinemia as opposed to normohomocysteinemia (80.76% versus 50%, p=0.005) [16].

According to the study conducted by Mundu et al., patients following an exclusively vegetarian diet showed elevated levels of serum homocysteine. The difference was statistically significant among diabetic patients with microvascular complications (Chi-square=5.812 at a significance level of 0.5, p=0.015) [17]. Abraham et al., in their study, showed that no significant difference exists between the serum homocysteine levels of vegetarians and non-vegetarians [18]. However, Upadhyay et al., in their study, showed that elevated levels of serum homocysteine are observed in diabetic populations who follow a strict vegetarian diet [19]. Vitamin B12 deficiency, commonly seen in people who follow a vegetarian diet for a long time, is responsible for increased homocysteine levels. Hence, this data can be used to deduce that vitamin B12 and folate supplementation in these patients can be employed to reduce the risk of hyperhomocysteinemia.

Limitations

As our study included only 92 subjects, which is a relatively small sample size, the limitation may have affected the generalizability of the findings to the general population. Hence, further prospective studies with larger populations are needed to better clarify the role of homocysteine in predicting microvascular complications. The study was conducted in a single medical center, which may limit the external validity of the results. Multicenter studies are required to validate our findings across different healthcare settings and geographic locations.

As low GFR independently raises the level of serum homocysteine, a study including populations with retinopathy and neuropathy, excluding nephropathy, needs to be undertaken to prove the vascular endothelial dysfunction of homocysteine leading to microvascular complications independent of low GFR.

## Conclusions

The major reason behind disability and death worldwide is diabetes mellitus. Individuals with poorly managed blood sugar can experience long-term consequences such as diabetic retinopathy, diabetic neuropathy, and diabetic nephropathy. This study was able to draw a significant association between high levels of plasma homocysteine and the microvascular complications, with a statistically significant difference in serum homocysteine values among the two groups, with a p-value of <0.001. A p-value of <0.05 was considered significant.

This was solidified by the multiple logistic regression analysis, which confirmed the established trend and identified hyperhomocysteinemia as a measurable risk factor for the development of all microvascular complications, diabetic nephropathy, retinopathy, and neuropathy. Additionally, there were noticeable differences in homocysteine levels between diabetic patients with microvascular complications and diabetic patients without microvascular complications, suggesting that homocysteine may be a useful biomarker for diabetic microangiopathy. The cutoff for serum homocysteine is taken as 11.8 µmol/L with a sensitivity of 83% and a specificity of 87% as a novel predictor of microvascular complications in type 2 diabetes mellitus patients.
